# Migration of Whooper Swans and Outbreaks of Highly Pathogenic Avian Influenza H5N1 Virus in Eastern Asia

**DOI:** 10.1371/journal.pone.0005729

**Published:** 2009-05-28

**Authors:** Scott H. Newman, Samuel A. Iverson, John Y. Takekawa, Martin Gilbert, Diann J. Prosser, Nyambyar Batbayar, Tseveenmyadag Natsagdorj, David C. Douglas

**Affiliations:** 1 EMPRES Wildlife Unit, Infectious Disease Group, Emergency Centre for Transboundary Animal Diseases, Animal Production and Health Division, Food & Agriculture Organization of the United Nations, Rome, Italy; 2 Wildlife Conservation Society, Field Veterinary Program, Bronx, New York, United States of America; 3 United States Geological Survey, Western Ecological Research Center, Vallejo, California, United States of America; 4 United States Geological Survey, Patuxent Wildlife Research Center, Beltsville, Maryland, United States of America; 5 Wildlife Science Conservation Center of Mongolia, and Ornithological Laboratory, Institute of Biology, Mongolia Academy of Sciences, Ulaanbaatar, Mongolia; 6 Ornithological Laboratory, Institute of Biology, Mongolia Academy of Sciences, Ulaanbaatar, Mongolia; 7 United States Geological Survey, Alaska Science Centre, Juneau, Alaska, United States of America; University of Arizona, United States of America

## Abstract

Evaluating the potential involvement of wild avifauna in the emergence of highly pathogenic avian influenza H5N1 (hereafter H5N1) requires detailed analyses of temporal and spatial relationships between wild bird movements and disease emergence. The death of wild swans (*Cygnus* spp.) has been the first indicator of the presence of H5N1 in various Asian and European countries; however their role in the geographic spread of the disease remains poorly understood. We marked 10 whooper swans (*Cygnus cygnus*) with GPS transmitters in northeastern Mongolia during autumn 2006 and tracked their migratory movements in relation to H5N1 outbreaks. The prevalence of H5N1 outbreaks among poultry in eastern Asia during 2003–2007 peaked during winter, concurrent with whooper swan movements into regions of high poultry density. However outbreaks involving poultry were detected year round, indicating disease perpetuation independent of migratory waterbird presence. In contrast, H5N1 outbreaks involving whooper swans, as well as other migratory waterbirds that succumbed to the disease in eastern Asia, tended to occur during seasons (late spring and summer) and in habitats (areas of natural vegetation) where their potential for contact with poultry is very low to nonexistent. Given what is known about the susceptibility of swans to H5N1, and on the basis of the chronology and rates of whooper swan migration movements, we conclude that although there is broad spatial overlap between whooper swan distributions and H5N1 outbreak locations in eastern Asia, the likelihood of direct transmission between these groups is extremely low. Thus, our data support the hypothesis that swans are best viewed as sentinel species, and moreover, that in eastern Asia, it is most likely that their infections occurred through contact with asymptomatic migratory hosts (e.g., wild ducks) at or near their breeding grounds.

## Introduction

Highly pathogenic avian influenza H5N1 (hereafter H5N1) has been circulating in avian populations since 1996 after it first emerged in eastern Asia and spread to large parts of Asia, Europe, Africa, and the Middle East. Mortality events involving large numbers of migratory birds first gained attention during spring 2005 with outbreaks occurring at Qinghai Lake, People's Republic of China (hereafter China), where >6,000 wild birds are reported to have died [1], [2], and during late summer 2005 in western China, northern Mongolia, Kazakhstan, and adjacent areas of Russia, where significant numbers of migratory bird mortalities also were confirmed [3]–[5]. It was widely hypothesized that these events indicated the onset of migratory bird involvement in the trans-continental spread of H5N1 [6] and viruses isolated from bar-headed geese (*Anser indicus*) and whooper swans (*Cygnus cygnus*) at these outbreak locations have become references to determine the genetic origins of viruses isolated in subsequent outbreaks [7]–[9]. However, despite the confirmation of H5N1 in numerous wild bird carcasses, surveillance of live birds has not elucidated a reservoir among free-ranging migratory species [e.g.], [10,11] and considerable uncertainty remains concerning the role of migratory birds in the perpetuation and geographic spread of H5N1 [12], [13].

The migratory bird die-offs in the spring of 2005 at Qinghai Lake and in northern Mongolia during the summer of 2005 raised concerns about the propensity of wild birds to become infected in one location and then translocate H5N1 over large distances when they migrate [14]–[16]. In the autumn & winter 2005–2006, >700 dead wild birds were recovered in 13 countries in Western Europe when only 4 countries had concurrent poultry outbreaks [3], [17]. The high level of biosecurity at European poultry farms, the broad spatial distribution of the outbreak locations, and the outbreak timing which coincided with temperature drops in the Black Sea region [18] have been used to suggest that infected wild birds carried virus hundreds of kilometers to the locations where the mortalities were documented [19], [20]. Swan moralities have drawn particular attention in relation to the role wild birds and H5N1 transmission, because swans are conspicuous species that are easily detected on the landscape and because of the large number of countries reporting outbreaks affecting swans [21]. For example, migratory whooper swans were among the first wild bird species documented with H5N1 in Mongolia and western China [3], [5], [17] and the only known instance of transmission of H5N1 directly from wild birds to humans occurred when villagers in Azerbaijan died after harvesting feathers from scavenged swan carcasses [3]. In Europe, most swan mortalities have involved mute swans (*Cygnus olor*), which are not generally considered a migratory species, although whooper swans, which are migratory, also have been affected [4], [5].

Experimental inoculation trials indicate that immunologically naïve swans are highly susceptible and die soon after exposure to H5N1 [22]–[23]. However, the onset of clinical illness in swans may be delayed for several days from the time of infection, during which time virus is shed [22] making swans potentially capable of concurrently transmitting virus while migrating [23]. There are no direct data available to determine the capacity for migration by swans after infection with H5N1, although at least one field study has suggested that migratory performance is impaired among Bewick's swans (*Cygnus columbianus bewickii*) infected with low pathogenicity avian influenza [24]. Moreover, although swan deaths have been the first indicator for the presence of H5N1 in numerous localities, disease-associated mortality does not imply that they play a central role in H5N1 virus transmission [6]. In this study, we used remote sensing to document the movements of whooper swans marked with GPS transmitters in eastern Asia. We tracked the movements of 10 whooper swans captured during the post-breeding molt period in northeastern Mongolia to wintering grounds in eastern China and on the Korean Peninsula. We evaluated these movements in relation to habitat type, potential interactions with domestic poultry, and the locations and timing of past H5N1 outbreaks in order to better understand their role in disease transmission. We predicted that if species like whooper swans, which are known to be highly susceptible to lethal infection, contracted H5N1 directly from poultry, then a majority of outbreaks involving such species should have been detected at times and in habitat types where their potential for exposure to poultry is highest. Alternatively, if the outbreaks affecting highly susceptible migratory waterbird species occurred at times and in habitats when the potential for direct exposure to poultry is low or nonexistent, it would provide evidence that the disease was contracted from a source other than poultry. We also evaluated the timing and location of outbreaks involving poultry in relation to the annual cycle of migratory waterbirds and predicted that if migratory waterbirds play an important role in transmitting virus to poultry, then the frequency of outbreaks affecting poultry should be higher during the winter period when migrants are present and that there should be a close spatial association with outbreak locations. Few studies have used direct ecological data to evaluate the transmission risk factors associated with H5N1 affected species, and our study was uniquely suited to infer the role of whooper swans in transmission of H5N1 in eastern Asia, a region where H5N1 is endemic and outbreaks persist in multiple areas including China and the Korean Peninsula.

## Methods

### Capture and marking

**Figure 1:**
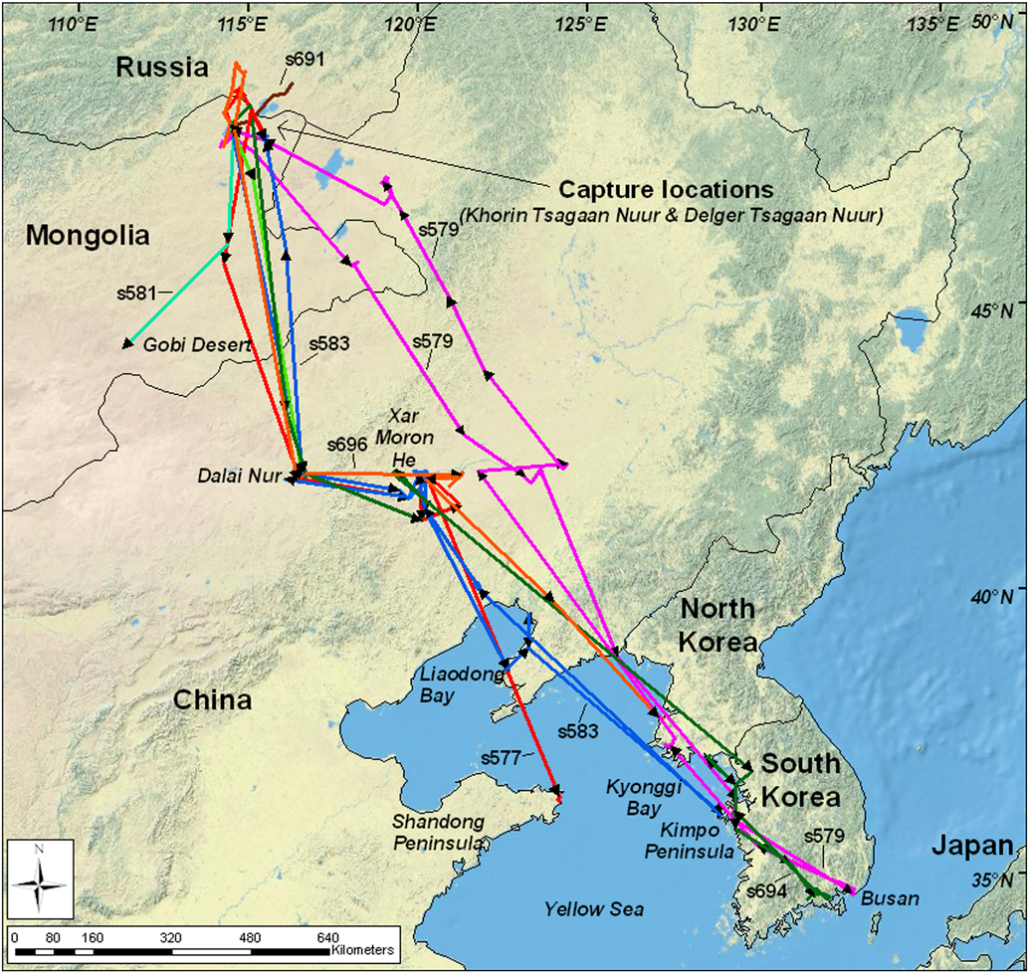
Map of the study area including satellite transmitter-marked whooper swan migratory routes.

The breeding range of whooper swans in eastern Asia extends from the Russian tundra south to the desert zone wetlands of China and Mongolia [25]. Evidence from banding and satellite tracking programs suggest little intermixing of Central Asian Flyway and the East Asian Flyway whooper swan populations [26]. In this study, we trapped whooper swans on two lakes in the East Asian Flyway, Khorin Tsagaan Nuur (49.66°N, 114.61°E) and Delger Tsagaan Lake (49.71°N, 114.58°E) in northeastern Mongolia (Figure 1) and tracked them for one annual cycle (1 August 2006–31 July 2007). Captures were made during wing molt, when swans are flightless, by paddling in kayaks and driving birds from water on to land and then corralling them. We drove many more birds to land than those that were captured and we believe that the birds captured are representative of the larger swan population in the area as there were 4–5 smaller lakes within 5–10 km and the total population of swans in this group of lakes totaled between 12,000–15,000 individuals in various stages of molt. We fit 10 with Argos-GPS solar-powered Platform Terminal Transmitters (PTTs; Microwave Telemetry Inc., Columbia, MD, USA). The 70 g transmitters (<1% swan mass), were equipped with internal receivers, solar panels, temperature and voltage sensors, and external antennae. They were attached dorsally with 1.4 cm wide woven tubular Teflon ribbon (Bally Ribbon Mills, Bally, PA, USA), with one length threaded within another for double-strength and knots secured by super glue (Henkel Loctite Corp., Rocky Hill, CT, USA).

Tracheal and cloacal swab samples were taken from each swan and stored at 4°C before being frozen in liquid nitrogen (maximum 3 hours post collection). Initial field diagnostic tests were performed using Flu Detect Antigen Test strips (Synbiotics Corp., Kansas City, MO, USA). Samples were shipped to the Southeast Poultry Research Laboratory (U. S. Department of Agriculture, Athens, Georgia), where they were screened for influenza *A* virus following standard testing protocols [27].

### Satellite telemetry locations

Transmitters were programmed to collect GPS locations at 2 -hr intervals throughout the 24 hr day and transmit signals to Argos satellites every 65 s for an 8 -hr on-cycle, followed by a 48 -hr off-cycle to facilitate solar recharging. Data were recovered from the Argos Data Collection and Location System (CLS America Inc., Largo, MD, USA) via receivers aboard polar-orbiting weather satellites. CLS calculated PTT location estimates that were derived from the perceived Doppler-effect shifts in transmission frequency during a satellite overpass. The accuracy of each location was rated by class. Class G locations indicated that the position was a GPS location with mean accuracy ±18.5 m. Conventional Argos location classes 0, 1, 2, and 3 indicated the location was derived from ≥4 transmissions, with accuracy >1000 m, 350–1000 m, 150–350 m, and ≤150 m, respectively. Location classes A (3 transmissions) and B (2 transmissions) are not assigned accuracy estimates by CLS, and location class Z indicates that no locations were obtained.

We compiled and validated our Doppler-derived location data using the Argos Filter Algorithm (D. Douglas, Version 7.03, http://alaska.usgs.gov/science/ biology/ spatial/). The filtering algorithm flags improbable locations based on user-defined distance and velocity thresholds. We used the algorithm to compile two datasets for analysis. The first dataset included one location per duty cycle, based on the highest-accuracy location class. Quality of signals was judged first on the basis of location class (G>3>2>1>0>A>B), and then by indices of residual frequency error. Our primary interest for compiling a dataset with one location per duty cycle was to evaluate broad-scale migration routes and movement chronology. For finer scale spatial analyses, we constructed a comprehensive dataset that included all GPS locations that were Class 1 or higher.

### Environmental data layers

To relate swan movements to landscape features and potential risk factors associated with H5N1 transmission we integrated our telemetry locations with digital thematic maps using ArcGIS, Version 9.2 (Environmental Systems Research Institute Inc., Redlands, California, USA). For habitat features we used MODerate-resolution Imaging Spectroradiometer (MODIS)/Terra Land Cover Classification, distributed by the Land Processes Distributed Active Archive Center, located at the United States Geological Survey Center for Earth Resources Observation and Science (http://LPDAAC.usgs.gov). The MODIS/Terra Land Cover Classification contains multiple classification schemes, the primary of which identifies 17 classes defined by the International Geosphere-Biosphere Programme (IGBP). We used a University of Maryland modification of the IGBP scheme (Land Cover Type 2), which we compiled into 4 land cover categories: (1) natural vegetation, including barren or sparsely vegetated areas; (2) water; (3) cropland or cropland mosaic; and (4) urban.

**Figure 2:**
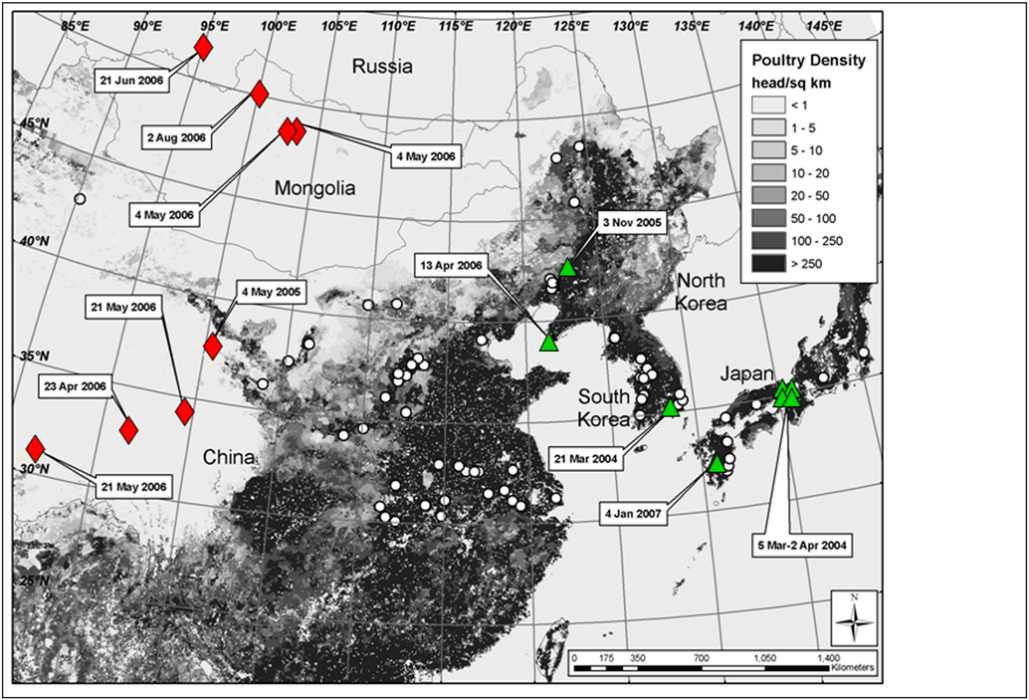
Poultry density and H5N1 outbreak locations [circles (poultry), diamonds (migratory waterbirds), and triangles (wild non-waterbirds)] in eastern Asia (2003–2007).

Poultry density information was obtained from the United Nations Food and Agriculture Organization (FAO) via the Geonetwork (http://www.fao.org/geonetwork). Methodology and sources of the estimates are described in the FAO's Gridded Livestock of the World [28]. Briefly, for each country the most recent available livestock census data were converted into densities to produce “observed” data and then disaggregated based on statistical relations with environmental variables in similar agro-ecological zones to produce “predicted” poultry distributions. The files were disseminated in raster format (0.0833-degree resolution), with pixel values representing predicted densities of poultry (head/km^2^; Figure 2).

Information about H5N1 outbreaks were obtained from the EMPRES Database [4] for the period 10 December 2003–8 March 2007 (Figure 2). We defined a rectangular study area for our analyses bounded to the northeast at 70°N, 80°E and the southwest at 25°N, 140°E. The study area included the breeding range of whooper swans in the East Asian Flyway, the full seasonal range of movements by the PTT-marked whooper swans, and regions of potential overlap with whooper swans from the Central Asian Flyway. Variables associated with each H5N1 outbreak included: date, location (country, administrative region, locality, latitude and longitude), reliability of the field veterinarian's diagnosis and the laboratory approach that was used to confirm viral subtype, and whether the outbreak occurred in wild or domestic animals. We restricted our analyses to records considered reliable by FAO EMPRES Program and the World Organization for Animal Health (OIE). We further categorized outbreaks involving wild birds as primarily affecting waterbirds (ducks, geese, swans, cormorants, grebes, or gulls) or being limited to non-waterbird species (crows, magpies, raptors). These wild bird groups are hereafter described using the terms “waterbirds” and “non-waterbirds” to distinguish them from domestic species.

### Whooper swan movements and H5N1 risk factors

Evaluation of the chronology and distribution of the marked-swans allowed us to identify post-breeding, migratory stopover, wintering, spring staging, and breeding areas, as well as distance from northerly breeding areas to the southernmost extent of the wintering range. Experimental studies have indicated a 1–5 d interval before the onset of clinical signs and 2–4 d duration of virus shedding for whooper swans experimentally inoculated with H5N1 [22]. Therefore, we also estimated the rate and distance traveled within such intervals during both the fall (southern) and spring (northern) migration as an indicator of the maximum distance that a swan could theoretically carry H5N1 at these times.

FAO-OIE confirmed H5N1 outbreaks were summarized by location and date. To evaluate seasonality of outbreak events we challenged a null hypothesis of equal probability of an outbreak for wild and domestic birds for all dates by comparing likelihood ratios during the whooper swan breeding and post-breeding periods, fall migration, winter period, and spring migration. For spatial evaluation, we used the Intersect Point feature in Hawth's Tools (Hawth's Analysis Tools for ArcGIS; Available: http://www.spatialecology.com/htools) to extract the habitat type and interpolated poultry density at each H5N1 outbreak location. Similar analyses were performed to determine the proportion of PTT-marked whooper swan locations occurring in each habitat type in relation to the underlying poultry density. We then evaluated trends in the occupancy patterns of croplands and urban areas through the annual cycle of the PTT-marked swans. To evaluate temporal variation in the possibility for direct contact between whooper swans and poultry, we weighted each PTT-marked swan equally and calculated weekly average poultry exposure estimates throughout the annual cycle.

## Results

### Capture and transmitter performance

All ten PTT-marked whooper swans were adults and tested negative for avian influenza virus. We received 5459 locations during the first year of tracking: 1 August 2006–31 July 2007 (Table 1). The period during which signals were received from transmitters ranged from one week to the full year with confirmation of at least one bird being killed by gunshot in Mongolia. The total number of locations obtained per swan (mean±SE) was variable (545.9±98.1 locations) and was influenced primarily by the duration over which signals were received (138.0±39.9 d). Overall, signal quality was very high with 89.2% of all locations being class 1 or higher, and 84.4% being a GPS fix with precision of ±18.5 m (Table 1).

**Table 1:** Satellite transmitter performance summary.

ID	Date of last transmission	Total locations	Location class*							
			G	3	2	1	0	A	B	Z
s577	24-Dec-2006	670	583	6	6	19	2	27	22	5
s579	21-Jul-2007	1009	809	13	12	12	6	65	88	4
s580	26-Aug-2006	191	160	10	3	4	2	5	6	1
s581	9-Oct-2006	514	445	5	13	18	8	12	8	5
s583	15-Jul-2007	790	607	18	20	20	7	44	70	4
s584	11-Aug-2006	82	72	0	0	1	2	2	5	0
s691	13-Sep-2006	269	222	6	5	7	2	16	10	1
s692	9-Oct-2006	451	375	8	11	9	11	16	19	2
s694	1-Mar-2007	931	854	2	4	14	19	24	12	2
s696	4-Dec-2006	552	480	1	5	11	16	15	22	2
	Total number of locations	5459	4607	69	79	115	75	226	262	26
	Percentage by location class		84.4%	1.3%	1.4%	2.1%	1.4%	4.1%	4.8%	0.5%

* Location classes are an indication of precision. Class G indicates a GPS fix with precision ±18.5 m. Conventional Argos location classes 3, 2, 1, and 0 indicate the location was obtained with 4 messages and precision ≤150 m , 150–350 m, 350–1000 m, and >1000 m, respectively. Location classes A (3 messages) and B (2 messages) do not have precision estimates and class Z indicates an unstable location solution.

### Migration chronology and routes

We divided the annual cycle of marked whooper swans into four stages on the basis of the area, scale of their movements, and arrival and departure dates. These were the: 1) breeding and post-breeding period (1 May to 2 October), 2) fall or southern migration (3 October–18 November), 3) non-breeding or winter period (19 November–20 February), and 4) spring or northern migration (21 February–30 April). The dates and annual cycle stages we used were appropriate for our sample population, but we recognize differences in chronology for whooper swans migrating to other breeding areas, particularly more northerly breeding areas, as well as the possibility for interannual variation in migration timing.

All ten swans were tracked during the post-breeding period (Table 2). Departure from the post-breeding area was documented for seven swans, five of which had complete fall migration histories (s577, s579, s583, s694, s696) and two that had partial histories (s581, s692). Three swans were tracked throughout the winter period (s579, s583, s694), while partial winter movement histories were available for the other two (s577, s696). Spring migration and breeding season movements were recorded for two swans (s579, s583).

**Table 2:** Migration chronology and transmission history summary.

	Transmitter ID								
	s577	s579	s580	s581	s583	s691	s692	s694	s696
Date marked (2006)	30-Jul	4-Aug	4-Aug	4-Aug	4-Aug	3-Aug	1-Aug	4-Aug	3-Aug
Date departed post-breeding area (2006)	27-Sep	24-Sep	–	8-Oct	6-Oct	–	5-Oct	26-Sep	14-Oct
Date arrived on winter area (2006)	6-Nov	8-Nov	–	–	15-Dec	–	–	22-Nov	12-Nov
Date departed winter area (2007)	–	21-Feb	–	–	21-Feb	–	–	20-Feb	–
Date arrived on breeding area	–	7-Apr	–	–	23-May	–	–	–	–
Total days monitored	147	351	22	66	345	41	69	209	123
Number of days with locations	119	220	22	64	169	37	65	160	98
Distance from capture location to southern most point (km)	1515	2000	–	–	1230	–	–	1950	1480

Table does not include s584, for which messages were not received >7 d after release.

**Figure 3:**
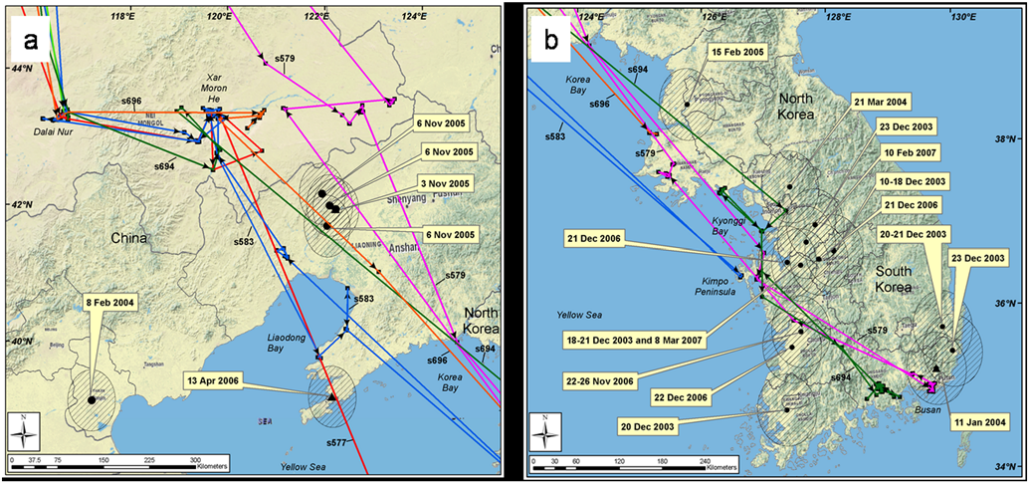
Whooper swan movements (2006–2007) and proximity to HPAI outbreak locations (2003–2007) affecting poultry (circles) and wild non-waterbirds (triangles) in (a) northeastern China and (b) the Korea Peninsula.

Departure dates from post-breeding areas ranged from 24 September–8 October 2006, with a mean of 2 October (Table 2). Four of five swans with complete fall migration histories used similar routes across the Gobi Desert to Dalai Nuur in Nei Mongol Province, China (s577, s583, s694, s696; Figure 1). Among these, three were observed to continue south from Dalai Nuur to Xar Moron He, a river that flows southeast into Xi Liao He, and then across Liaoning Province to the Korean Peninsula (s583, s694, s696; Figure 3). The fourth swan, s577, used a more westerly route across Liaoning Province, before traveling across Liondong Bay and the northern part of the Yellow Sea to Shandong Peninsula, China. In contrast, s579 followed a more easterly route from the breeding area, bypassing both Dalai Nuur and Xar Moran He. However, as was the case with s583, s694, and s696, it too wintered on the Korean Peninsula. Wintering areas could not be determined for s692, which began along the same route as the majority of swans toward Dalai Nuur, or (s581), which headed southwesterly across the Gobi Desert, before their transmissions ceased.

Arrival dates on the wintering area ranged from 6 November–15 December and averaged 19 November (Table 2). Swan s577 spent the remainder of its transmission period, until 24 December, on the southeastern tip of Shandong Peninsula, approximately 225 km northeast of Qingdao, China. On the Korean Peninsula, two swans (s579, s694) traveled as far south as Nakdong Estuary, Busan in Kyongsang-namdo Province, South Korea. The last location received for s696 was on 3 December in South Hwanghae Province, 80 km southwest of Pyongyang, North Korea. Only two locations were received for s583 between 25 November 2006 and 23 February 2007, both low quality locations in the vicinity of Kimpo Peninsula in Chungcheong Province, South Korea.

Estimated departure dates for spring migration ranged from 20–21 February for the three swans (s579, s583, s694) that still wore functioning PTTs at the end of the winter period (Table 2). The last signal for s694 was recorded on 23 February from a diked tidal flat on the Kimpo Peninsula, about 100 km southwest of Seoul, South Korea. Locations were received with greater frequency during spring migration for s583, which allowed monitoring during the spring migration and breeding period. The last transmission for s583 was recorded near its original capture site in Dornond Aimag, Mongolia on 15 July. Similarly, s579 was tracked all the way back to the breeding grounds, returning to Khorin Tsagaan Nuur in Dornond Aimag, with the last signal received on 13 June.

All seven of the whooper swans that were tracked during migration exhibited the potential for long distance dispersal in a short (<5 d) time period. The rate of travel was particularly rapid during fall. For example, s577 spent 58 d at different wetlands within 90 km of its capture site and then flew 430 km south across the Gobi Desert during an 18 hr period (23.9 km/hr: Table 3). After spending approximately 5 wk in the vicinity of Dalai Nuur and Xar Moron He, s577 flew 707 km south in a 31 hr period (22.8 km/hr) to the tip of Shandong Peninsula. Similar movements were recorded for the other swans, the fastest being a 559 km movement by s579 across the Gobi Desert in a 12 hr period (46.6 km/hr) on 19 October, and the longest a 946 km movement in 39 hr (24.3 km/hr) on 7–8 November from Xi Liao He, China to Kyonggi Bay, South Korea by s694. The mean duration was 48.2±7.1 d during the fall migration, with the shortest recorded interval being 29 d (s694) and the longest 70 d (s583). The migration rate during spring was slower for the two PTT-marked swans that returned to their breeding areas. Duration estimates were 46 and 92 d, for s579 and s583, respectively, yielding mean of 69.0±23.1 d. Significant spring migration movements included a 456 km flight in ≤30 hr (15.2 km/hr) on 21–22 May by s583 from Dalai Nuur across the Gobi Desert to northeastern Mongolia, and a 674 km flight in 120 hr (5.6 km/hr) from Korea Bay to Xi Liao He by s579. Total distance from breeding to the southernmost extent of wintering areas averaged 1640±148 km.

**Table 3:** Long distance migration movements recorded for whooper swans during fall 2006 and spring 2007.

ID	Migration period	Date	Distance (km)	Time (hr)	Rate (km/hr)	Description of movement
s577	Fall	27 Sep	464	18.1	25.6	Capture area across the Gobi Desert to Nei Mongol Province, China
	Fall	5–6 Nov	707	31.0	22.8	Xar Moron He to the Shandong Peninsula
s579	Fall	19 Oct	559	12.0	46.6	Eastern Mongolia across the Gobi Desert to Nei Mongol Province, China
	Fall	3–5 Nov	780	78.1	10.0	Nei Mogol Province, China to Kyonggi Bay on the Korean Peninsula
	Spring	14–19 Mar	674	120.0	5.6	From Korea Bay to Xi Liao He
s581	Fall	8–9 Oct	518	42.0	12.3	From the capture area across the Gobi Desert
s583	Fall	5–7 Oct	722	71.8	10.1	Capture area across the Gobi Desert to Dalai Nuur
	Spring	21–22 May	456	30.0	15.2	Dalai Nuur to the breeding area in northeastern Mongolia
s694	Fall	26–28 Sep	723	72.0	10.0	Capture area across the Gobi Desert to Dalai Nuur
	Fall	7–8 Nov	946	39.0	24.3	Xi Liao He to Kyonggi Bay on the Korean Peninsula
s696	Fall	15–17 Oct	723	44.8	16.1	Capture area across the Gobi Desert to Dalai Nuur
	Fall	9–12 Nov	743	82.0	9.1	Dalai Nuur to Yongsang-dong 80 km southwest of Pyongyang

### Reported HPAI outbreaks in eastern Asia

We included 95 FAO-OIE confirmed H5N1 outbreak events involving wild and domestic birds in eastern Asia in our analyses. These included 79 cases involving poultry, eight in which wild waterbirds were affected, and eight that were restricted to non-waterbird species. A majority of cases involving poultry did not specify whether Galliformes (e.g. chickens, quails and turkeys) or Anseriformes (e.g. domestic ducks and geese) were the primary species affected. Therefore, we evaluated poultry as a single group rather than comparing these potentially different categories. The incidents involving waterbirds affected several species, including: whooper swans, bar-headed geese, Pallas's (great black-headed) gulls (*Larus ichthyaetus*), brown-headed gulls (*Larus brunnicephalus*), ruddy shelducks (*Tadorna ferruginea*), great cormorants (*Phalacrocorax carbo*), goosander (*Mergus merganser*), Eurasian wigeon (*Anas penelope*), black-necked crane (*Grus nigricollis*), and several unspecified species of grebe (*Podiceps spp*.), egret or heron (family Ardeidae), and “wild ducks” not identified to species (family Anatidae).

Two of the eight outbreaks involving mostly waterbirds also affected small numbers on non-waterbird species. Also, official reports of H5N1 outbreaks involving non-waterbird species were somewhat imprecise with regard to species and indicated that crows and magpies (family Corvidae) and hawks (family Accipitridae), including one mountain hawk eagle (*Spizaetus nipalensis*) were affected. It should be noted that detection of wild bird mortality events is more likely to occur when large numbers of individuals perish at once, when there are fewer scavengers, in habitats that are monitored as wildlife parks, protected wetlands or RAMSAR sites, in habitats frequented by tourist or birders, or in locations with greater human densities. As well, large white birds such as whooper swans are more likely to be observed dead in the environment than small passerine species. Along the migration routes of whooper swans in this study, there is some passive surveillance, annual distribution and population counts are performed, and it is more likely that a mortality event would be detected in the southern part of the migration pathway as this coincides with greater human densities and important birds and conservation habitats regularly monitored.

**Figure 4:**
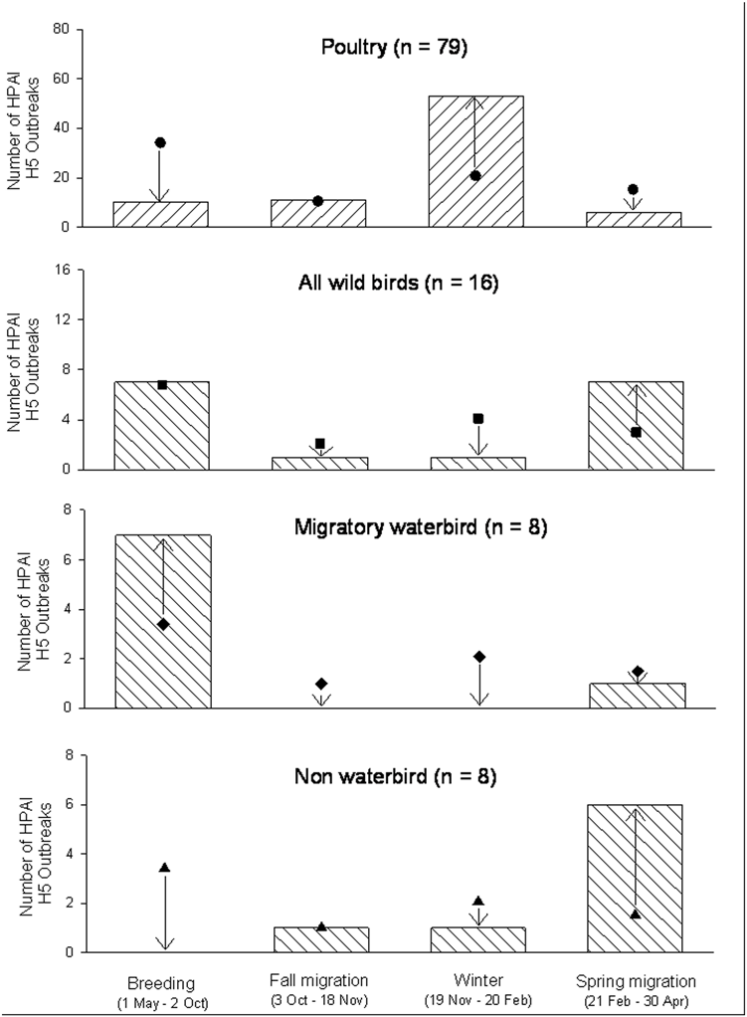
Frequency of poultry and wild bird H5N1 outbreak events in relation to season as determined by whooper swan annual cycle stage. Circles, squares, diamonds, and triangles represent the expected number of outbreaks in each of bird sector, bars represent the actual number of outbreaks, and arrows up/down indicate if there were greater than or less than expected numbers of outbreaks, respectively.

With respect to seasonality, seven of the eight (87.5%) H5N1 outbreaks involving waterbirds occurred during the whooper swan breeding period and likelihood ratio tests indicated an over representation of occurrence during this period relative to the number of exposure days (G = 7.1; df - = 1; P = 0.01; Figure 4). Among these, five of seven occurred shortly after arrival to breeding areas (late April and May) and in one instance, late during the spring migration period, just before arrival on the breeding grounds. For non-waterbird species, six of the eight (75.0%) outbreak events occurred during the whooper swan spring migration period, which likelihood ratio testing also indicated was non-random with respect to the number of exposure days (G = 13.2; df = 1; P<0.001). For poultry, 53 of the 79 (67.0%) outbreaks occurred during the whooper swan wintering period, which was a greater proportion than other seasons on the basis of likelihood ratios and exposure days (G = 59.2; df = 1; P<0.001; N = 79).

With respect to habitats, all eight of the outbreak events involving waterbirds occurred in natural vegetation (grasslands), where poultry densities were estimated to be <1 head/km^2^. For the non-waterbird species, outbreaks were recorded in urban (N = 7) and cropland (N = 1) habitat types, where poultry densities averaged 337 (±114 SE) head/km^2^ and ranged from 0–874 head/km^2^. The outbreaks involving poultry occurred in a wider variety of habitats, with most categorized as urban (N = 38) or cropland (N = 29), but several were associated with areas of natural vegetation (N = 12). Estimated poultry densities at the location of outbreaks in poultry averaged 1020±118 head/km^2^ and ranged from 0–6070 head/km^2^.

### Whooper swan movements in relation to habitat, poultry and H5N1 outbreaks

PTT-marked whooper swans almost exclusively occupied habitats comprised of natural vegetation types or water (98.4%) during the breeding and post-breeding periods, when most location estimates were characterized as grassland (81.7%). During fall migration, predominant occupancy continued to be in natural areas (48.9%), but there was an increasing diversity of habitat occupancy, including water (26.8%) and croplands (23.9%). For the winter period as a whole, habitat categories were spread evenly among natural vegetation types (25.5%), water (29.4%), croplands (21.1%), and urban (24.0%) areas. However, there was also a pattern wherein use of croplands and urban areas tended to increase from the early to late winter, eventually constituting >90% of all locations just prior to spring migration. During the early stages of spring migration use of croplands was high suggesting that whooper swans likely moved into agricultural fields to forage before departing for breeding areas.

Not surprisingly, potential interactions between PTT-marked whooper swans and poultry mirrored habitat use patterns. Estimates for the mean poultry density at whooper swan locations ranged from 0–5 head/km^2^ during the post-breeding period (Table 4). Estimates increased during fall migration, peaking at 212 head/km^2^ during the late fall, and remained high throughout the winter period, ranging from 174–3460 head/km^2^. The increased use of croplands by whooper swans during late winter also was reflected in an increase in poultry exposure during late winter, with the highest poultry densities recorded from mid-February to mid-March. Poultry density estimates at swan locations were high during early spring (1760 head/km^2^), and then decreased to 0–4 head/km^2^ during the breeding season.

**Table 4:** Mean weekly exposure of whooper swans to poultry by annual cycle stage.

Stage	Minimum poultry density (head/km^2^)	Maximum poultry density (head/km^2^)
Post-breeding and breeding	0.0	4.9
Fall migration	15.4	212.0
Winter	174.0	3460.0
Spring migration	0.0	1760.0

**Figure 5:**
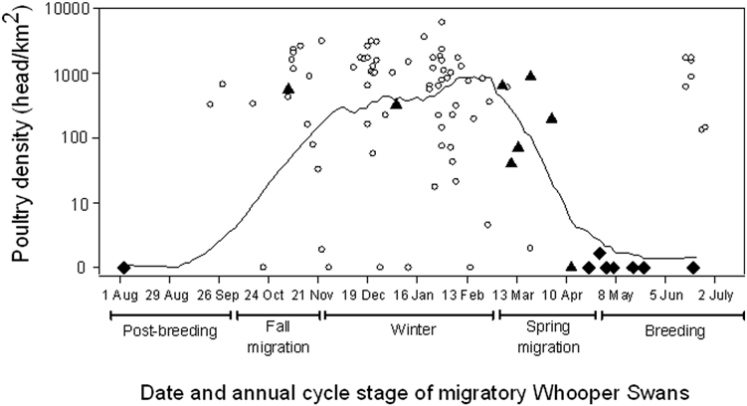
Relationships among migratory whooper swan movements, the timing of H5N1 outbreaks, and the density of poultry in eastern Asia. Solid line represents mean poultry density at whooper swan locations through the annual cycle. Circles (poultry), diamonds (migratory waterbirds), and triangles (wild non-waterbirds) indicate the timing and underlying poultry density at each H5N1 outbreak event.

The correlation between potential exposure to poultry and the timing of H5N1 incidents in waterbirds in eastern Asia was very low (Figure 5). None of the H5N1 outbreaks involving waterbirds occurred during winter, when the potential for interaction with poultry and probability of direct transmission from poultry to migratory waterbirds was predicted to be highest. Instead, seven of the eight (87.5%) waterbird mortality events occurred when waterbirds had already returned to breeding areas.

Among poultry, most outbreaks occurred during winter when migratory waterbirds were present. However, there was a strong tendency for outbreaks affecting poultry to occur in urban and cropland areas with high poultry density, rather than in areas with abundant natural vegetation and less poultry, the more frequently used wintering habitat of whooper swans and other waterfowl. Moreover, the frequency of outbreaks involving poultry did not increase during late winter and the early spring migration period when whooper swans were observed to spend more time in urban and cropland habitats where there is a higher potential exposure to poultry.

## Discussion

Understanding the role that migratory waterbirds birds play in the ecology and transmission of H5N1 requires the integration of habitat data, seasonal movement chronology and routes, domestic poultry production information, dates and locations of H5N1 outbreak events, and analyses at both temporal and spatial levels. For many regions in which H5N1 is endemic and migratory waterbirds are suspected of disease introduction or dispersal, such basic data are often lacking. We predicted that if highly susceptible migratory waterbirds like whooper swans contracted H5N1 directly from poultry, then an association should be apparent with respect to the timing and location of outbreaks. This was clearly not the case.

Although outbreaks of H5N1 involving poultry were numerous in East Asian Flyway portions of China and the Korean Peninsula from 2003–2007, very few cases involving non-waterbird species were reported in close association with poultry outbreaks although non-waterbird outbreaks did occur amongst a background of moderate poultry density (Figure 2). This is in contrast to migratory waterbird outbreaks that were reported primarily to the north and west of poultry production centers, often in sparsely populated areas (Figure 2). With respect to the timing of H5N1 outbreaks involving migratory waterbirds, most outbreaks in eastern Asia occurred during late spring and early summer coinciding with an energetically demanding period associated with migration over hundreds to thousands of kilometers followed by reproduction. These two important life cycle phenomena likely make birds more susceptible to diseases, as they are both immunologically and metabolically compromised.

Additionally, wild bird outbreak sites were characterized by grassland habitats where poultry density was very low (<1 head/km^2^) and the timing was, in nearly all instances, several weeks to months after the initial estimated departure from wintering areas where poultry density was high. In this respect, the situation in eastern Asia differs from that documented in Europe, where numerous swan mortalities were detected in proximity to locations where poultry are present [5]. Thus, while in Europe it might be argued that swans are directly infected from poultry (i.e., spillover), and they may be responsible for local movement of virus regionally, followed by the potential to transmit virus back to poultry (i.e., spillback), this does not appear to have been the case in eastern Asia.

In eastern Asia, the distribution and timing of outbreaks strongly suggests the involvement of an intermediary vector. The sites where H5N1 outbreaks affecting migratory waterbirds were documented tend to be locations of potential convergence by variety of migratory species from both the East-Asian Australasian and Central Asian flyways. We speculate that the likely explanation for virus introduction was via asymptomatic co-mingling wild ducks capable of circulating virus within their populations without succumbing to illness. Evidence for such a mechanism has been demonstrated [29], whereby virus isolates that are non-pathogenic in wild and domestic ducks replicate and transmit efficiently to naïve contacts. Under this paradigm, wild ducks would contract H5N1 from poultry and then translocate it over great distances as they migrate, and whooper swans or other migratory waterbirds that die from H5N1 in the region would have contracted the disease via secondary contact [30] including exposure to fresh faeces or contaminated water or vegetation. We speculate that ducks are more resilient to H5N1 HPAI virus infection, and therefore, more likely to be virus carriers compared to swans or geese because they constitute a more prominent natural reservoir for avian influenza viruses circulating in both the Palaearctic and Nearctic. Ducks out-number both swan and geese globally, in both the wild and domestic bird sectors, making ducks important as a host. Hence, from an evolutionary ecology perspective, ducks are more likely to have developed resistance to the pathogenic effects of avian influenza viruses over time, or may exhibit immunity due to cross-protection from exposure to other avian influenza strains. Specifically related to H5N1 HPAI, we may have actually observed adaptation in ducks given the virus emerged in Guangdong province in the People's Republic of China in 1996, the global epicentre of domestic ducks.

In the East-Asian Australasian and Central Asian flyways, ruddy shelduck (*Tadorna ferruginea*), mallard (*Anas platyrhynchos*), common teal (*A. crecca*), northern pintail (*A. acuta*), and Eurasian wigeon (*A. penelope*) are the numerically dominant duck species migrating between breeding areas in Siberia and Mongolia and wintering areas in Japan, China, Southeast Asia, and India [31] where outbreaks in poultry have been most common. Unfortunately, these species have not been adequately sampled in surveillance programs to determine if they are potentially carriers of H5N1, and it is also possible that one or more of these duck species is an asymptomatic or periodic shedder making surveillance less likely to detect virus upon sampling.

Another potential explanation for the lack of correspondence between the timing of outbreak events affecting whooper swans and their periods of exposure to poultry is that they did not succumb to disease immediately after infection. Whooper swans used croplands with increasing frequency during late winter, a pattern that also has been observed in Bewick's swans [32]. In eastern Asia, this led to whooper swans moving into habitats with high poultry densities (1760 head/km2), at a time when peak grazing of domestic ducks occurs in rice fields [33]. However, this finding was apparent only for a small sample of individuals and would also imply a capacity to suppress illness during migration. This seems highly improbable given what is known about the ecologic immunology of H5N1 in migratory birds [34]. Conversely, Kalthoff et al. [23], found that one adult mute swan with avian influenza virus antibodies acquired from a previous natural infection with low pathogenicity avian influenza, survived after exposure to highly pathogenic H5N1. This suggests that pre-existing antibodies may offer some protection to swans and potentially other species of birds exposed H5N1, recognizing that viral pathogenicity and exposure dose may also be important factors determining survival. Furthermore, experimental inoculation studies [22]–[23] demonstrate that it can take several days for clinical symptoms to occur in H5N1 exposed wild bird species, a time frame that could allow for migrating whooper swans to move relatively large distances (Table 3).

During 2003–2007, H5N1 poultry outbreaks in eastern Asia occurred year round, but peaked during winter. Because the southern extent of migratory ranges of whooper swans and other migratory waterbirds bring them into proximity to high poultry density areas during the non-breeding season, one would anticipate frequent H5N1 outbreaks in both poultry and waterbirds if direct transmission among poultry to waterbirds was occurring. However, our analysis indicated a lower number of confirmed waterbird outbreaks than expected relative to the number of poultry exposure days. Rice-duck agriculture in eastern Asia is of such prominence that it provides for the world epicentre of domestic mallards (700 million birds in China alone), and although less wetland associated, domestic geese populations exceed 300 million birds [15]. Increasing poultry production and densities, increased market and trade movements, coupled with cold stress and overcrowding of domestic birds which intensifies in the fall peaking in the winter, may all contribute to an increased H5N1 viral load, environmental contamination, and regional dispersal of virus. The build-up of an H5N1 repository in faecal material in agricultural fields or along shorelines of water bodies could explain the fact that non-waterbird species outbreaks in eastern Asia during 2003–2007 occurred primarily during late February through April in urban locations that had a mean poultry density of 337 head/km^2^.

From a disease spread and transmission standpoint, laboratory studies have demonstrated that whooper swans experimentally inoculated with H5N1 can shed significant virus (>4log^10^ EID_50_/mL) for up to 4 d before the onset of clinical signs [22], a period of time in which swans marked in this study travelled distances of 400–800 km when migrating. As well, mute swans have demonstrated a much slower than expected transmission and infection rates within infected populations [19] and a genuine risk of spatial redistribution of virus via swan movements can be identified. However, our data also indicate that migration between wintering and breeding areas is prolonged, with many stopover sites being used by migrating whooper swans. Few wild bird carcasses were recovered along migratory routes and poultry outbreaks did not track migratory waterbird movements. The data collected on whooper swan migration ecology and behaviour, coupled with the timing of H5N1 outbreaks and the relative poultry density at the outbreak locations, suggest the species has been a victim, rather than vector of disease transmission in eastern Asia. However, many unanswered questions remain concerning the mechanisms whereby H5N1 arrives at migratory waterbird breeding areas. We believe that our findings contribute to the limited knowledge that is currently available regarding ecological correlates to H5N1 infections affecting migratory birds, and highlight the need for results to be amalgamated from studies in different regions, using a variety of research tools, on multiple species groups. It is only through such a process that we will fully understand relationships between wild birds and the perpetuation and spread of H5N1.
